# Method to represent the distribution of QTL additive and dominance effects associated with quantitative traits in computer simulation

**DOI:** 10.1186/s12859-016-0906-z

**Published:** 2016-02-06

**Authors:** Xiaochun Sun, Rita H. Mumm

**Affiliations:** Department of Crop Sciences and the Illinois Plant Breeding Center, University of Illinois at Urbana-Champaign, 1102 S. Goodwin Ave., Urbana, IL 61801 USA; Present address: Dow AgroSciences, Indianapolis, IN USA; GeneMax Services, Urbana, IL 61802 USA

**Keywords:** Dirichlet process, Gaussian mixture model, Genetic architecture, Meta-analysis, QTL effects

## Abstract

**Background:**

Computer simulation is a resource which can be employed to identify optimal breeding strategies to effectively and efficiently achieve specific goals in developing improved cultivars. In some instances, it is crucial to assess *in silico* the options as well as the impact of various crossing schemes and breeding approaches on performance for traits of interest such as grain yield. For this, a means by which gene effects can be represented in the genome model is critical.

**Results:**

To address this need, we devised a method to represent the genomic distribution of additive and dominance gene effects associated with quantitative traits. The method, based on meta-analysis of previously-estimated QTL effects following Bennewitz and Meuwissen (J Anim Breed Genet 127:171–9, 2010), utilizes a modified Dirichlet process Gaussian mixture model (DPGMM) to fit the number of mixture components and estimate parameters (i.e. mean and variance) of the genomic distribution. The method was demonstrated using several maize QTL data sets to provide estimates of additive and dominance effects for grain yield and other quantitative traits for application in maize genome simulations.

**Conclusions:**

The DPGMM method offers an alternative to the over-simplified infinitesimal model in computer simulation as a means to better represent the genetic architecture of quantitative traits, which likely involve some large effects in addition to many small effects. Furthermore, it confers an advantage over other methods in that the number of mixture model components need not be known *a priori*. In addition, the method is robust with use of large-scale, multi-allelic data sets or with meta-analyses of smaller QTL data sets which may be derived from bi-parental populations in precisely estimating distribution parameters. Thus, the method has high utility in representing the genetic architecture of quantitative traits in computer simulation.

**Electronic supplementary material:**

The online version of this article (doi:10.1186/s12859-016-0906-z) contains supplementary material, which is available to authorized users.

## Background

Computer simulation is a resource which can be employed to identify optimal breeding strategies to effectively and efficiently achieve specific goals in developing improved cultivars [1, 2]. Once identified, optimal strategies can be incorporated in the development ‘process’ to facilitate maximal genetic gains and accelerate the breeding process [3]. Through computer simulation, innovative approaches heretofore not feasible without use of current genomic-based technologies can be specifically tailored to meet the need at hand. In some instances, it is crucial to assess *in silico*, the options as well as the impact of various crossing schemes and breeding strategies on performance for certain key agronomic traits such as grain yield. Such is the case with introgression (i.e. integration) of value-added traits by means of backcross breeding, the goal of which is the recovery of all the performance attributes of the elite variety or hybrid targeted for conversion along with the new genetic elements (e.g. genes, QTL, or transgenic events) associated with the value-added trait. For this, a means by which the gene effects for the performance attributes of the line to be converted can be modeled in the simulation is particularly advantageous, yet it requires an accurate depiction of the distribution of gene effects. Typically, recovery of the target line (i.e. recurrent parent) is estimated by the average proportion of genetic material carried through the backcrossing process and this estimate implicitly assumes that the many genes for key quantitative traits like grain yield are dispersed uniformly across the genome, each contributing only small effect. By including a more realistic representation of gene effects in the genome model to assess backcross breeding strategies, the means to most rapidly and effectively recover not only the germplasm *per se*, but the important genes contributing to performance of the variety or hybrid targeted for conversion, can be considered in evaluating strategies and approaches.

Bennewitz and Meuwissen [4] explored the distribution of additive and dominance effects of identified QTL (quantitative trait loci) from three F2 populations of pigs evaluated for 34 meat quality and carcass traits, recognizing the value of modeling these effects, some of which are large, over use of the infinitesimal model which assumes an infinite number of QTL each with small effect. Capitalizing on the large number of QTL studies, Bennewitz and Meuwissen [1] conducted a meta-analysis of published QTL mapping data across traits to infer the distribution of additive QTL effects as well as dominance coefficients, fitting a Gaussian mixture model (GMM). The idea of utilizing GMM is based on the notion that various QTL and associated genes fall into a number of classes of different-sized effects. The merit of employing GMM is its flexibility with different combinations of mixtures of normals leading to different shapes of the distribution.

In the finite mixture model, the number of components *K* must be pre-specified. The value could be determined based on some specific information or criteria, such as the Akaike information criterion or the Bayesian information criterion. This requirement, frequently encountered in parametric statistics, can be sidestepped by introducing a nonparametric Dirichlet process which assumes an infinite number of components. The Dirichlet process is defined as a random process by which a sample drawn is a discrete distribution; it can be considered a ‘distribution over distributions’ and has been used widely in the field of population genetics to explain population structure [5, 6].

Also desiring to capitalize on the large number of QTL studies, we took the meta-analysis concept a step further. We devised a method to represent the genomic distribution of additive and dominance gene effects associated with quantitative traits, which utilizes a modified Dirichlet process Gaussian mixture model (DPGMM) [7] to fit the number of mixture components and estimate parameters. As a departure from traditional DPGMM which only models QTL effects, we modified the model to be able to accommodate both QTL effects and their respective variances. Utilizing previously-identified QTL for a number of quantitative traits in maize, the modified Dirichlet process implements a Chinese Restaurant Process (CRP) to assign component (cluster) membership and uses Gibbs sampling to update conditional posterior distributions. Our purpose in devising this method was to facilitate representation of the genetic architecture of grain yield and other key traits for use in computer simulations to optimize breeding strategies for multiple trait introgression (see Sun and Mumm [8] for an example of utilization of DPGMM output). Trait introgression involves backcross breeding and, therefore, bi-parental populations with no more than two potential alleles for a given locus; this was the primary scenario we intended to model. However, we also explored whether the method would be pertinent to other modeling activities that may involve estimation of breeding value (such as for choice of parents) or prediction of performance based on priors [9], scenarios which could involve multi-allelic populations in various crop or animal species. Besides use in computer simulation, an accurate depiction of the QTL effects could contribute to a better understanding of the overall genetic architecture contributing to variation of expression of a particular trait of interest [10].

## Methods

### Description of DPGMM and priors

To begin, we modeled the distribution of additive QTL effects and dominance coefficients using mixtures of normal distributions, namely GMM [11] . The goal was to assign genetic effects to different mixture components. Two latent variables were introduced: 1) the total number of mixture components (cluster size, *K*) and 2) the assignment of *i*^*th*^ QTL effect to components (cluster indicator, *c*_*i*_ ∈ {1, …, *K*}). The GMM model was modified to accommodate the standard errors of QTL effects:

1 $$ p\left({y}_i\Big|{\lambda}_1,\dots, {\lambda}_K\right)\sim {\displaystyle \sum_{k=1}^K{\pi}_kN\left({y}_i;{\mu}_k,{\sigma}_k^2+{\tau}_i^2\right)}, $$

where *y*_*i*_ is the *i*^*th*^ observed QTL effect, *τ*_*i*_ is the known standard error of *i*^*th*^ effect which is calculated from the QTL analysis, and *λ*_*k*_ = {*π*_*k*_, *μ*_*k*_, *σ*_*k*_^2^} is the *k*^*th*^ parameter set, where variables *π*_*k*_, *μ*_*k*_ and *σ*_*k*_^2^ are the mixing proportion, mean and variance of the *k*^*th*^ mixture component, respectively. The DPGMM can be formulated hierarchically as follows [7]:

2 $$ \begin{array}{l}p\left({y}_i\Big|{c}_i,\boldsymbol{\Lambda} \right)\kern0.5em \sim \kern0.5em N\left({y}_i;{\mu}_{c_i},{\sigma}_{c_i}^2+{\tau}_i^2\right)\\ {}{c}_i\Big|{\pi}_{1:K}\kern0.5em \sim \kern0.5em  Discrete\left({\pi}_1,{\pi}_2,\dots {\pi}_K\right)\\ {}\left({\mu}_k,{\sigma}_k^2\right)\kern0.5em \sim \kern0.5em {G}_0\\ {}{\pi}_1,{\pi}_2,\dots {\pi}_K\kern0.5em \sim \kern0.5em  Dirichlet\left(\alpha /K,\dots, \alpha /K\right)\\ {}\boldsymbol{\Lambda} =\left\{{\lambda}_1,{\lambda}_2,\dots {\lambda}_K\right\}\end{array} $$

where *G*_0_ is a joint prior distribution for (*μ*_*k*_, *σ*_*k*_^2^) and mixing proportions *π*_1 : *K*_ are drawn from a symmetric Dirichlet distribution with concentration parameter *α*. Conditional on the mixing proportions, the latent indicator variables *c*_*i*_’s were sampled from a discrete distribution, specifically a multinomial distribution, and the prior for the *c*_*i*_ in model [2] could be written as a probability conditional on **c**_− *i*_ [12]:

$$ p\left({c}_i=k\Big|{\mathbf{c}}_{-i},\alpha \right)=\frac{n_{-i,k}+\alpha /K}{n-1+\alpha }, $$

where *n*_− *i*,*k*_ is the number of effects, not including *y*_*i*_ that are linked with class *k*. And as *K* goes to infinity (*K* → ∞), the limits of the prior for the *c*_*i*_ reach the following:

3 $$ p\left({c}_i\Big|{\mathbf{c}}_{-i},\alpha \right)=\left\{\begin{array}{cc}\hfill \frac{n_{-i,k}}{n-1+\alpha}\hfill & \hfill {c}_i=k,{n}_{-i,k}>0\hfill \\ {}\hfill \frac{\alpha }{n-1+\alpha}\hfill & \hfill \forall i\ne {i}^{\hbox{'}},{c}_i\ne {c}_{i\hbox{'}}\hfill \end{array}\right.. $$

where *i’* is the complement of the set *i*. As *K* → ∞, the Dirichlet distribution becomes a Dirichlet process in the limit [12, 13]. Thus, infinite limit of model [2] can be written as a DPGMM:

4 $$ \begin{array}{l}\kern2.5em {y}_i\kern0.75em \sim \kern0.75em N\left({y}_i\Big|{\theta}_i,{\sigma}_i^2\right)\\ {}\left({\mu}_i,{\sigma}_i^2\right)\kern0.75em \sim \kern0.75em G\\ {}\kern2.5em G\kern0.5em \sim \kern0.5em \mathrm{D}\mathrm{P}\left(\alpha, {G}_0\right),\end{array} $$

where *θ*_*i*_ ~ *N*(*μ*_*i*_, *τ*_*i*_^2^) is a nuisance parameter, *G* is a random discrete distribution drawn from DP, and *G*_0_ was the base distribution, which specified the joint prior distribution of (*μ*_*i*_, *σ*_*i*_^2^). Given that the regular choice of priors for the mean and variance of the Gaussian are normal and inverse gamma distributions, respectively, conjugate joint priors *N*(*μ*_*i*_; *μ*_0_, *σ*_0_^2^)* *IG*(*σ*_*i*_^2^; *r*_1_, *r*_2_) were chosen in the model in order to implement the following Gibbs Sampling.

### Gibbs Sampling

In Bayesian framework, unknown variables were sampled and updated from the conditional posterior distribution using Markov Chain Monte Carlo (MCMC) [14]. Considering the likelihood and priors in Formulae 3 and 4, the full joint posterior distribution can be written as follows:

5 $$ \begin{array}{l}p\left(\mathbf{c},\boldsymbol{\upmu}, {\boldsymbol{\upsigma}}^2\Big|\mathbf{y}\right)\propto {\displaystyle {\prod}_{i=1}^nN\left({y}_i;{\theta}_i;{\sigma}_i^2\right)}\pi \left({\theta}_i,{\mu}_i,{\sigma}_i^2,{c}_i\right)\kern17.25em \\ {}\kern5.25em ={\displaystyle {\prod}_{i=1}^nN\left({y}_i;{\theta}_i;{\sigma}_i^2\right)}N\left({\theta}_i;{\mu}_i;{\tau}_i^2\right)N\left({\mu}_i;{\mu}_0;{\sigma}_0^2\right)IG\left({\sigma}_i^2;{r}_1;{r}_2\right)p\left({c}_i\Big|{\mathbf{c}}_{-i},\alpha \right).\end{array} $$

Unobservables (**c**, **μ**, **σ**^2^) were repeatedly sampled and updated from their posteriors, conditional on all other variables. The Gibbs sampler was implemented as follows:

1. Initialization: Assign initial values for (*μ*_*k*_, *σ*_*k*_^2^) where *k* = 1 and *c*_*i*_ = 1, for *i* = 1 : *n*.
2. Update *θ*_*i*_: The conditional posterior distribution of *θ*_*i*_ was

$$ P\left({\theta}_i\Big| else\right)\propto N\left({y}_i;{\theta}_i;{\sigma}_k^2\right)N\left({\theta}_i;{\mu}_k;{\tau}_i^2\right)\propto N\left({\theta}_i;\frac{\frac{y_i}{\sigma_k^2}+\frac{\mu_k}{\tau_i^2}}{\frac{1}{\sigma_k^2}+\frac{1}{\tau_i^2}},\frac{1}{\frac{1}{\sigma_k^2}+\frac{1}{\tau_i^2}}\right) $$

3) Update cluster indicators *c*_*i* :_ The conditional posterior probabilities for *c*_*i*_ were:

$$ \begin{array}{l}P\left({c}_i=k\Big| else\right)\propto N\left({y}_i;{\theta}_i;{\sigma}_k^2\right)N\left({\theta}_i;{\mu}_k;{\tau}_i^2\right)p\left({c}_i\Big|{\mathbf{c}}_{-i},\alpha \right)\\ {}\kern5.75em =\frac{n_{-i,k}}{2\pi \sqrt{\sigma_k^2{\tau}_i^2}} \exp \left(-\frac{{\left({y}_i-{\theta}_i\right)}^2}{2{\sigma}_k^2}-\frac{{\left({\theta}_i-{\mu}_k\right)}^2}{2{\tau}_i^2}\right),\end{array} $$

$$ \begin{array}{l}P\left({c}_i=K+1\Big| else\right)\propto \alpha {\displaystyle \iint N\left({y}_i;{\theta}_i;{\sigma}_{K+1}^2\right)}N\left({\theta}_i;{\mu}_{K+1};{\tau}_i^2\right)N\left({\mu}_{K+1};{\mu}_0;{\sigma}_0^2\right)IG\left({\sigma}_{K+1}^2;{r}_1;{r}_2\right)d{\mu}_{K+1}d{\sigma}_{K+1}^2\ \\ {}\kern7em \propto \alpha {\displaystyle \int N\left({y}_i;{\theta}_i;{\sigma}_{K+1}^2\right)IG\left({\sigma}_{K+1}^2;{r}_1;{r}_2\right)d{\sigma}_{K+1}^2}{\displaystyle \int N\left({\theta}_i;{\mu}_{K+1};{\tau}_i^2\right)}N\left({\mu}_{K+1};{\mu}_0;{\sigma}_0^2\right)d{\mu}_{K+1}\\ {}\kern7em =\frac{\alpha }{2\pi}\frac{{r_2}^{r_1}}{\varGamma \left({r}_1\right)}\frac{\varGamma \left({r}_1+\frac{1}{2}\right)}{{\left(\frac{1}{2}{\left({y}_i-{\theta}_i\right)}^2+{r}_2\right)}^{r_1+{\scriptscriptstyle \frac{1}{2}}}}\sqrt{\frac{1}{\left({\tau}_i^2+{\sigma}_0^2\right)}} \exp \left(-\frac{{\left({\theta}_i-{\mu}_0\right)}^2}{2\left({\tau}_i^2+{\sigma}_0^2\right)}\right)\kern4em \end{array} $$ where *Γ*(.) is the gamma function. Note that constant $\frac{1}{n-1+\alpha }$ was omitted in both probabilities and (*μ*_*K* + 1_, *σ*_*K* + 1_^2^) were unknown and needed to be integrated out to leave *c*_*i*_ as the only variable to be estimated from the Markov Chain. The Dirichlet Process was represented via the CRP [15]. Effects were assigned to either currently holding cluster(s) or a new cluster based on the above probabilities. If a new cluster was chosen, then the cluster size was increased, *i.e. K* + 1 → *K*. In case of *n*_− *i*,*k*_ = 0, the *k*^*th*^ cluster was eliminated and the cluster indicators were decreased by one, *i.e. K* → *K* − 1.

4) Resample and update (*μ*_*k*_, *σ*_*k*_^2^) suggested by Formulae 2 as per Neal [12] as follows:

$$ \begin{array}{l}P\left({\mu}_k\Big|{\theta}_i\in {k}^{th} cluster, else\right)\propto {\displaystyle \prod_{i=1}^{n_k}N\left({\theta}_i;{\mu}_k;{\tau}_i^2\right)}N\left({\mu}_k;{\mu}_0;{\sigma}_0^2\right)\\ {}\kern10.5em \sim N\left({\mu}_k;\frac{{\displaystyle \sum_{i=1}^{n_k}\frac{\theta_i}{\tau_i^2}}+\frac{\mu_0}{\sigma_0^2}}{{\displaystyle \sum_{i=1}^{n_k}\frac{1}{\tau_i^2}}+\frac{1}{\sigma_0^2}},\frac{1}{{\displaystyle \sum_{i=1}^{n_k}\frac{1}{\tau_i^2}}+\frac{1}{\sigma_0^2}}\right)\end{array} $$

$$ \begin{array}{l}P\left({\sigma}_k^2\Big|{y}_i\in {k}^{th} cluster, else\right)\propto {\displaystyle \prod_{i=1}^{n_k}N\left({y}_i;{\theta}_i;{\sigma}_k^2\right)}IG\left({\sigma}_k^2;{r}_1;{r}_2\right)\\ {}\kern10.5em \sim IG\left({\sigma}_k^2;{r}_1+\frac{n_k}{2},\frac{1}{2}{\displaystyle \sum_{i=1}^{n_k}{\left({y}_i-{\theta}_i\right)}^2+{r}_2}\right),\end{array} $$

where *n*_*k*_ is the number of effects associated with the *k*^*th*^ mixture component. The derivations of the fully conditional posterior distributions are detailed in the Appendix.

5) Repeat Steps 2 to 4.

Gibbs sampler was implemented with 100,000 iterations of the MCMC to update conditional posterior distributions. The first 80,000 samples were discarded as burn-in and the rest of the 20,000 samples were used to construct joint posterior distribution. The hyper-parameters in Algorithm 5 were set to be *α* = 0.05, *r*_1_ = 1, *r*_2_ = 0.01, *μ*_0_ = 0, *σ*_0_^2^ = 0.01. Among hyperparameters, alpha was empirically set to 0.05 based on the simulation results. (Note: The larger the magnitude of alpha, the higher the probability of a large number of clusters.) Convergence was checked by inspection of negative log-likelihood plots. After the burn-in period, when the MCMC converges to the stationary distribution, sampled parameters were collected to form the posterior distribution. We employed posterior means for estimating the mean and variance $\left({\widehat{\mu}}_k,{\widehat{\sigma}}_k^2\right)$ and posterior modes for estimating *ĉ*_*i*_, which was further used to infer ${\widehat{\pi}}_k$. The Bayesian confidence interval (BCI), which is the counterpart of the confidence interval in frequentist statistics, was defined as posterior probability that the parameter lies within the interval:

$$ {\displaystyle \underset{-\infty }{\overset{A}{\int }}p\left(\boldsymbol{\Lambda} \Big|Y\right)d\boldsymbol{\Lambda} ={\displaystyle \underset{B}{\overset{\infty }{\int }}p\left(\boldsymbol{\Lambda} \Big|Y\right)d\boldsymbol{\Lambda} =\alpha /2}}, $$

where *α* is the significance level. Instead of analytically estimating the confidence interval, the confidence interval for $\left({\widehat{\mu}}_k,{\widehat{\sigma}}_k^2\right)$ was numerically estimated from quartiles of posterior distribution.

### Demonstration of method performance with simulated data

To demonstrate the performance of the proposed method, two simulated data sets were processed. Simulation I facilitated evaluation of model performance given complete data. It was generated from three GMMs with respective means -1, 0 and 1 and variances of 0.360, 0.640 and 0.040, respectively. A total of 150 simulated QTL effects were uniformly distributed (mixing proportion was 1/3) to the three components. This data set represents the case wherein all true QTL are known.

Simulation II facilitated evaluation of model performance on a truncated distribution. Truncation points were arbitrarily set to ± 0.1. The incomplete data set was intended to represent the common situation with QTL mapping data wherein all genetic effects are not detected, especially those with effects of near-zero magnitude. A truncated Gaussian mixture with two mixture components was simulated. Zero mean was assigned to both components. The first mixture component had mixing proportion *π*_1_ and variance *σ*_1_^2^ of 0.8 and 0.023, respectively; the second mixture component had *π*_2_ and *σ*_2_^2^ of 0.2 and 0.360, respectively. In both simulations, the standard error (SE) *τ*_*i*_ was generated from a uniform distribution [0, 0.01].

### Implementation with real data

The model was also applied using real data to derive the distributions of additive effects and dominance coefficients. Additive QTL effects were assembled from previous QTL mapping studies performed in corn [16–18] (see Table 1 for a list of traits and associated QTL for each data set, referred to as Data I, Data II, and Data III respectively). Messmer et al. [17] had evaluated recombinant inbred lines derived from a cross between two subtropical white dent maize lines to map genes controlling yield components and other traits [15], identifying 57 QTL in total which are included in Data I. Briggs et al. [16] had utilized a maize-teosinte backcross (BC_1_) population to explore genes controlling domestication and morphological traits such as plant architecture, primary tassel and lateral inflorescence, identifying 59 QTL in total which are included in Data II. Data III is derived from five maize QTL mapping studies involving segregating populations, all of which share a common parent B73, comprising a total of 101 quantitative trait loci including a) 11 QTL for kernel oil concentration mapped in an F_2_ population [19]; b) 15 QTL for root angle and plant height mapped in an F_2_ population [20]; c) 31 QTL for stalk digestibility and kernel composition mapped in a F_3_ population [21]; d) 6 QTL for stripe disease resistance mapped in an F_2_ population [22]; and e) 38 QTL for grain yield and yield components mapped in an F_3_ population under water-limited conditions [23]. All QTL mapping studies employed either composite interval mapping or multiple interval mapping to detect QTL. [18]. Furthermore, these QTL studies all reflect estimates of gene effects in bi-parental populations, which fit with the backcross breeding scenario we intended to simulate. The histograms of observed additive effects, with values represented in units of phenotypic standard deviations, are shown in Fig. 2 for all three data sets.

**Table 1:** QTL associated with various traits across four data sets. Data I, II, III, and IV were included in the analysis of QTL additive effects and Data III was used in the analysis of QTL dominance coefficients

Data sets	Traits	Number of QTL detected
Data I	Days to anthesis	12
	Anthesis-to-silking interval	8
	Grain yield	5
	Kernel number	7
	100-kernel weight	11
	Plant height	14
Data II	Branch number	2
	Cob diameter (teosinte)	4
	Culm diameter	1
	Cupules per rank	2
	Days to pollen	4
	Glume score	5
	Inflorescence length	2
	Lateral branch internode	3
	Lateral branch	2
	Lateral inflorescence branch	1
	Length of central spike	2
	Male spikelet length	3
	Mean lateral branch internode	2
	Number of barren nodes	1
	Number of tassel branches	5
	Percent staminate spikelets	3
	Plant height (teosinte)	6
	Prolificacy	2
	Ranks of cupules	3
	Tassel branching space length	5
	Tillering	1
Data III	Kernel oil concentration	11
	Root angle	10
	Plant height	5
	Dry matter digestibility (*in vitro)*	4
	Cell wall digestibility (*in vitro)*	3
	Neutral detergent fiber	4
	Acid detergent fiber	5
	Water-soluble carbohydrate	2
	Kernel oil content	4
	Kernel protein content	4
	Kernel starch content	5
	Stripe virus resistance	6
	Grain yield	3
	100-kernel weight	9
	Kernel number per ear	6
	Cob weight per ear	7
	Kernel weight per ear	3
	Ear weight	5
	Ear number per plant	5
Data IV	20-kernel weight	202
	Days to anthesis	403

To explore application with multi-allelic, large-scale data sets as an alternative to bi-parental QTL data sets, additive QTL effects were assembled from high-resolution genome-wide association studies (GWAS) with the maize NAM (Nested Association Mapping) population [18], data provided courtesy of Jason G. Wallace (Department of Crop and Soil Sciences, University of Georgia; email: jason.wallace@uga.edu). From the 41 traits in the data set, 2 were chosen to generate Data IV: ‘20-kernel weight’ (weight in grams of 20 seeds; yield component trait) and ‘days to anthesis’ (developmental trait) (Table 1). Only those significant single nucleotide polymorphisms (SNPs) featured in at least three resample inclusions were utilized to fit the distribution (i.e. 202 significant QTL for 20-kernel weight and 403 significant QTL for days to anthesis). The QTL had been detected through composite interval mapping or multiple interval mapping and the association mapping had been performed using the forward-regression genome-wide association method in TASSEL 4.1.32 [18]. Results were compared to those obtained with QTL mapping data sets from bi-parental populations to contrast multi-allelic vs. bi-allelic, number of QTL, power in detection of the QTL, and single-trait distributions vs. distributions representing multiple traits.

In addition, the distribution of dominance effects was explored. Dominance coefficients, which are defined as the ratio between the observed QTL dominance deviation and absolute value of QTL additive effects, were assembled from Data III. The absolute value of additive QTL effects was used because the sign of QTL effect only signifies which parent had contributed the favorable allele, not the true direction of specific additive effect. The SE for additive QTL effects and dominance coefficients was measured to take into account the experimental error. If logarithm (base 10) of the odds (LOD) scores for QTL were absent, standard errors were generated by taking sample standard deviation of effects from multiple experiments. The SE of Data II and of data from Dintinger et al. [22] incorporated in Data III were produced in this way, where only those QTL detected in at least two environments were included in the final dataset. For the rest of the studies, SEs were derived from LOD scores as suggested by Hayes and Goddard [24] or, in the case of the GWAS data sets, the SEs were determined from the sample errors of the discovered SNP effects [18]. Standard errors of dominance coefficients were estimated by the delta method suggested by Bennewitz and Meuwissen [1], assuming no covariance between additive and dominance effects. Specifically, $S{E}_{d/a}=\left(d/a\right)*\sqrt{{\left(\frac{S{E}_a}{a}\right)}^2+{\left(\frac{S{E}_d}{d}\right)}^2}$, where *SE*_*a*_ and *SE*_*d*_ were standard errors for additive effects *a* and dominance effects *d*, respectively.

Additive QTL effects from QTL mapping studies were scaled by their corresponding phenotypic standard deviations in order to combine data across traits. The phenotypic standard deviations were computed using raw data if available from the QTL study. Otherwise, the error variance and heritability of the trait were used to calculate the phenotypic standard deviation or, absent this, the range of phenotype values were used. Phenotypic range was assumed to be 8 standard deviations, which covers almost 100 % of the values, considering that most traits follow a normal distribution. We did not apply phenotypic standardization to the GWAS data since an abundance of data points were available. Since for the data sets from which dominance effects were generated, none of the above three conditions was fulfilled to obtain the phenotypic standard deviations for additive QTL effects, the additive effects from those five corn studies were not utilized in analyzing the distribution of additive QTL effects. Note that the scaling process was not necessary for dominance coefficients, because the phenotypic standard deviation canceled out in the *d/a* ratio.

Due to limited statistical power of QTL mapping studies [25], many QTL with near-zero effects were likely not detected in the published studies used in this analysis, which is effectively analogous to a truncation of the additive QTL effect distribution near zero. Faced with this issue, Bennewitz and Meuwissen [1] suggested a “doubling” process, given the assumption that the true QTL effects occur at the highest frequency around zero. To compensate for the ‘missing’ QTL effects, a doubling of the data was done to ensure that the mean of each mixture component is estimated at zero, preserving the characteristic greatest density at zero for each cluster. With doubling, both signs for the same QTL additive effect were created. For example, for *i*^*th*^ effect *y*_*i*_ with SE *τ*_*i*_, − *y*_*i*_ was added to the data with the same SE. The above procedure leverages the fact that absolute values of alternative homozygous genotypes at a QTL are the same by definition e.g. in bi-parental populations [26]. The “doubling” process was not applied to dominance coefficients, because most loci have observed effects around zero.

## Results and discussion

In this study, we employed a new method, namely DPGMM, to describe the distribution of QTL additive effects and dominance coefficients in the form of mixtures of normals. Although similar to the fitting of a mixture of normals using a modified expectation-maximization (EM) algorithm, this approach differs primarily in the way of dealing with cluster size (*K*). With DPGMM, the number of mixture components does not need to be specified (it is assumed to be infinite). In contrast, with use of a finite mixture model, the number of components needs to be preset and later decided under certain circumstances, or determined by some measure, e.g. Akaike information criterion or Bayesian information criterion. The optimum cluster size (*K*) will strike a balance between maximum data compression (assigning all data to one component) and maximum accuracy (allowing the number of clusters equal sample size).

In DPGMM, the Dirichlet process is represented by the CRP, wherein a data point is assigned either to a currently occupied mixture component with probability proportional to the number of data already held in that cluster or to a new cluster with probability proportional to the concentration parameter, *G*_0_. By the same token, with each iteration of Gibbs sampling, the cluster indicators are also updated along with parameters like the mean and variance. As such, DPGMM fits the data distribution and explores the potential number of mixture components simultaneously.

**Fig. 1:**
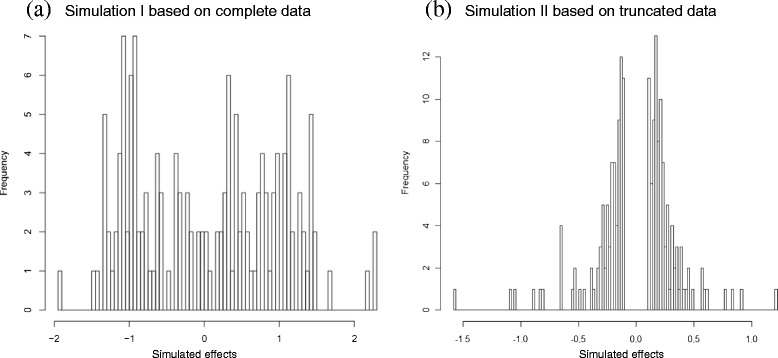
Histograms of simulated effects from Gaussian mixtures: **a** (*n* = 150) three components having mean of -1, 0 and 1, and variance of 0.36, 0.64 and 0.04, respectively, and equal mixing proportions for all three components; and (**b**) (*n* = 300) two components having zero means and variance of 0.023 and 0.36, respectively, and mixing proportions of 0.8 and 0.2, respectively. Distribution in (**b**) is truncated at points -0.1 and 0.1

To demonstrate the performance of DPGMM in fitting the distribution of QTL effects and to verify accuracy in estimating associated parameters of this distribution, two simulated data sets were processed. Simulation I was structured to represent the case wherein all true QTL are known i.e. complete data. Simulation I, which featured three components, resulted in a histogram of genetic effects from which it is difficult to infer the number of mixture components visually (Fig. 1a). In Simulation I, DPGMM clearly fitted the data to three clusters with estimated values close to true values for parameters involving the proportion of mixing among clusters, the mean and the variance of each mixture component (Table 2). DPGMM predicted accurately the mean and variance of Clusters 1 and 3, although missed assignments of cluster membership were observed. In contrast, the mixing proportion of Cluster 2 was estimated precisely; however, certain deviations from the true mean and variance were observed.

**Table 2:** True versus estimated (hat) parameters in Simulation I. *π*_*k*_ is the mixing proportion in the *k* ^*th*^ cluster, and *μ*_*k*_ and *σ*_*k*_^2^ are the mean and variance of *k* ^*th*^ mixture component, respectively. Values expressed in units of phenotypic standard deviation

	Cluster1	Cluster2	Cluster3
*π*_*k*_	0.333	0.333	0.333
${\widehat{\pi}}_k$	0.487	0.367	0.147
*μ*_*k*_	1.000	0.000	-1.000
${\widehat{\mu}}_k$	0.841	-0.673	-1.041
*σ*_*k*_^2^	0.360	0.640	0.040
${\widehat{\sigma}}_k^2$	0.312	0.303	0.012

Simulation II data, based on a truncated mixture of normals featuring two mixture components with zero mean, resemble a scenario common to QTL mapping wherein near-zero genetic effects were not detected. Simulation II produced a histogram with a pronounced gap around zero as expected in keeping with the data truncation (Fig. 1b). In Simulation II, parameters were estimated with accuracy, except for the variance of Cluster 1, which was estimated at 0.251 versus the true value of 0.023 (Table 3).

**Table 3:** True versus estimated (hat) parameters in Simulation II. *π*_*k*_ is the mixing proportion in the *k* ^*th*^ cluster, and *μ*_*k*_ and *σ*_*k*_^2^ are the mean and variance of *k* ^*th*^ mixture component, respectively. Values expressed in units of phenotypic standard deviation

	Cluster1	Cluster2
*π*_*k*_	0.800	0.200
${\widehat{\pi}}_k$	0.912	0.089
*σ*_*k*_^2^	0.023	0.360
${\widehat{\sigma}}_k^2$	0.251	0.382

Given a complete set of data, DPGMM could clearly assign membership to respective clusters with small prediction errors (Fig. 1a, Table 2). In the case of truncated data, DPGMM was still effective in predicting the correct number of mixture components and estimating the variance of components with greater variability; however, DPGMM was less effective in estimating variance of components with small differences among cluster members. As shown in Table 3, the deviation of estimated variance (0.251) from true value (0.023) was somewhat large and might be attributed to the loss of small-value data in the sample. This result is in accord with the conclusion of Bennewitz and Meuwissen that small effects could be missed easily with a mixture model [1].

Proceeding to real data, DPGMM was used as a method to fit a mixture of normals for which the number of components is not known, fitting additive effects and dominance coefficients based on previously published QTL mapping data for a number of quantitative traits in maize. In addition, DPGMM was used to fit additive effects estimated from high-resolution GWAS of the maize NAM population to compare distributions produced with large-scale, multi-allelic data sets involving a single trait with those obtained with meta-analysis of bi-parental, lower-resolution studies involving multiple traits. The fitted distributions are the outcome of applying the DPGMM method and these distributions could then become the basis for modelling the genetic architecture of quantitative traits in maize for computer-simulated explorations to identify optimal breeding strategies.

### Distributions of QTL additive effects

**Fig. 2:**
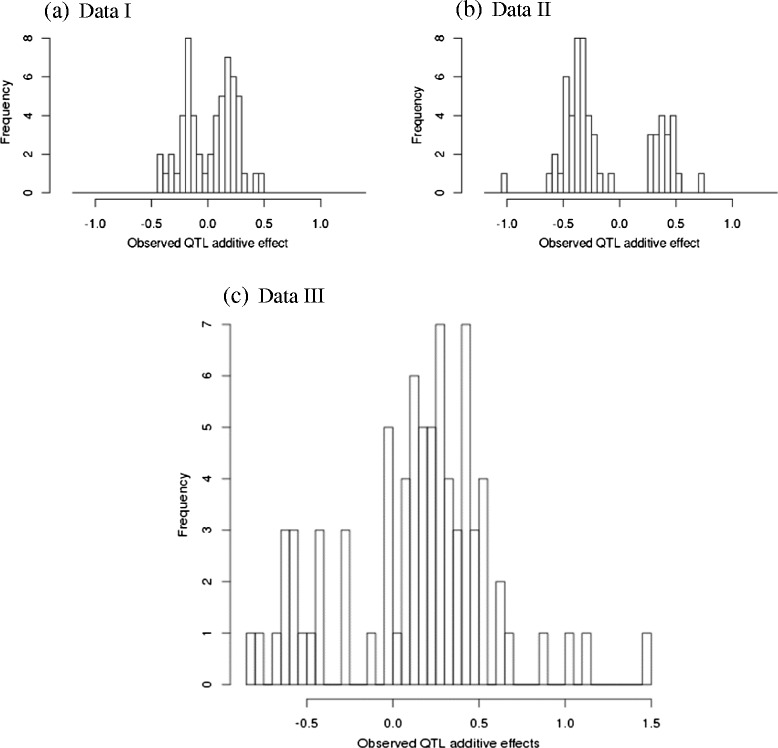
Histograms of observed QTL additive effects (expressed in units of phenotypic standard deviation): **a** Data I; **b** Data II; and **c** Data III

**Fig. 3:**
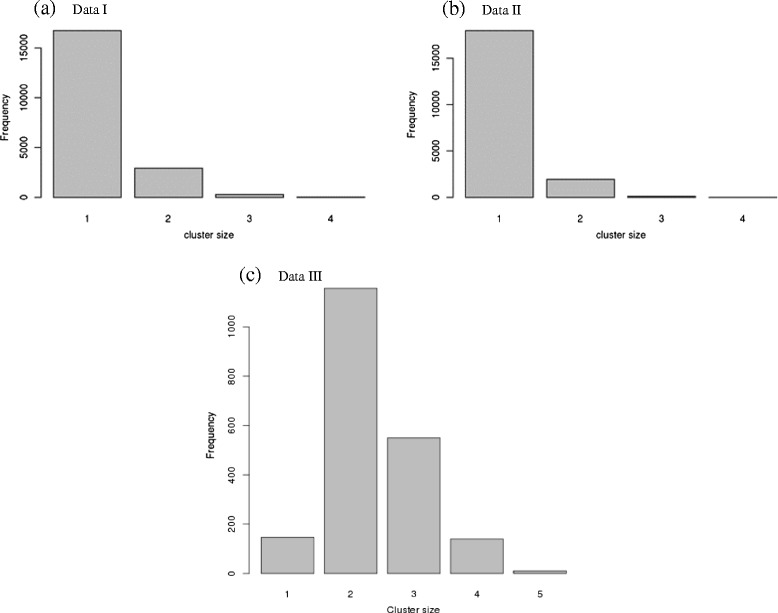
Histograms of the cluster number: **a** Data I; **b** Data II; and **c** Data III

Histograms of observed QTL additive effects expressed in units of phenotypic standard deviation were generated from the QTL mapping studies (Fig. 2). We noted that the histogram of Data II (Fig. 2b) resembled that observed with Simulation II (Fig. 1b), wherein near-zero effects were not included. With Data I, II, and III, it is difficult to infer the number of mixture components for additive effects visually from the histograms. However, the number of mixture components was inferred by the mode of posterior distribution with regard to cluster indicator *c*_*i*_. Frequency tables of cluster membership clearly suggested fitting all data to one cluster for Data I and Data II (Fig. 3); however, for Data III, two components are suggested to fit additive effects. This likely reflects the types of traits included in each meta-analysis. Data I contains primarily grain yield and yield component traits as well as some developmental traits. Data II is comprised of mainly domestication and morphological traits. Data III includes yield and yield component traits under drought stress as well as grain quality traits. Results suggest more similarity among the traits within Data I and within Data II, in contrast to Data III that was fitted with two clusters.

**Fig. 4:**
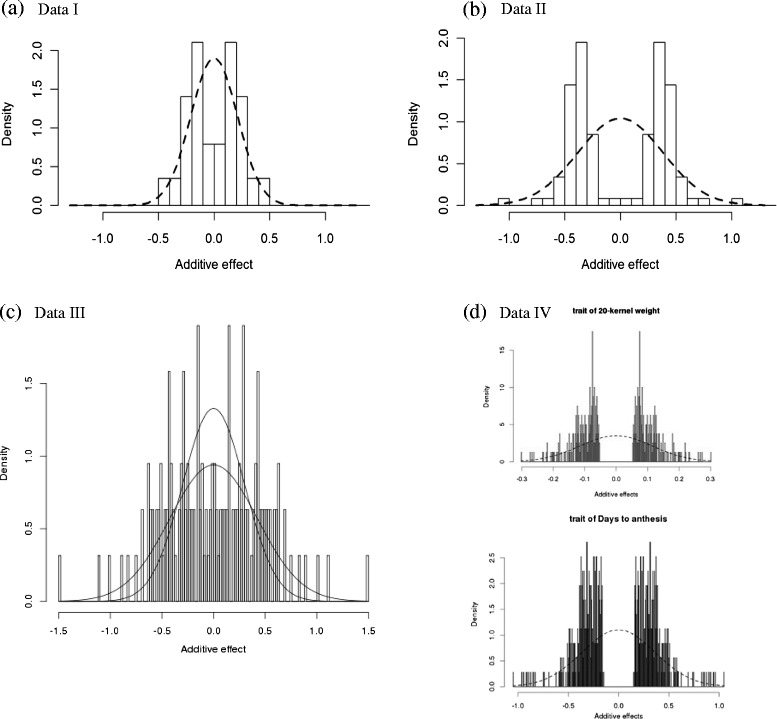
Fitted normal distributions to QTL additive effects (expressed in units of phenotypic standard deviation): **a** Data I; **b** Data II; **c** Data III; **d** Data IV featuring traits of 20-kernel weight and days to anthesis

For each of the three QTL mapping data sets, the fitted distribution was overlaid on the histogram of “doubled” data (Fig. 4a, b, c). The fitted distributions of additive effects all have zero mean; variances differed (Table 4). The range of the observed QTL effects was tight around the mean for Data I, whereas Data II showed some larger effects with absolute value nearing 1. With Data III, the range of observed effects was greater still, with the largest effects nearing 1.5 in absolute value; nonetheless, the variance of Data III additive effects was similar to that of the distribution of Data II. The variance was smallest for Data I comprised of mainly yield and yield component traits.

**Table 4:** Estimates (hat) of the mixing proportion in the *k* ^*th*^ cluster (*π*_*k*_), the cluster mean and variance (*μ*_*k*_ and *σ*_*k*_^2^ ) and Bayesian confidence interval (BCI) for parameters in the distribution of additive effects and dominance coefficients. Values expressed in units of phenotypic standard deviation

Data sets	Effect type	Estimated parameters	Posterior estimate	BCI	
				2.50 %	97.50 %
Data I	Additive	${\widehat{\sigma}}_1^2$	0.044	0.034	0.059
Data II	Additive	${\widehat{\sigma}}_1^2$	0.147	0.110	0.194
Data III	Additive	${\widehat{\pi}}_1$	0.880	0.451	0.973
		${\widehat{\pi}}_2$	0.120	0.006	0.572
		${\widehat{\sigma}}_1^2$	0.179	0.068	0.227
		${\widehat{\sigma}}_2^2$	0.107	0.008	0.200
	Dominance coefficient	${\widehat{\mu}}_1$	0.152	0.055	0.237
		${\widehat{\sigma}}_1^2$	0.329	0.193	0.542
Data IV	Additive				
20-kernel weight		${\widehat{\sigma}}_1^2$	0.013	0.011	0.015
Days to anthesis		${\widehat{\sigma}}_1^2$	0.131	0.118	0.146

Comparing the results obtained with the Data IV GWAS single-trait data sets, the data were fitted to one cluster for both 20-kernel weight and days to anthesis. Each fitted distribution was overlaid on the histogram of “doubled” data (as was done with the meta-data sets). Each distribution had an estimated mean of zero. The variance associated with 20-kernel weight was estimated as 0.013, with a narrow confidence interval, 0.011 to 0.015; the variance for days to anthesis was estimated as 0.131, with a narrow confidence interval, 0.118 to 0.146 (Table 4). The fitted distribution of 20-kernel weight based on 202 QTL shows a high degree of similarity to that for Data I which is comprised of 57 QTL for yield and yield component traits, maturity, and abiotic stress (Fig. 4). The fitted distribution of days to anthesis based on 403 QTL shows a high degree of similarity to that for Data II which is comprised of 59 QTL for domestication and morphological traits (Fig. 4). Compared to distributions from meta-analyses, the GWAS data sets resulted in greater precision as displayed in the relatively smaller BCI’s for the estimates of variance; the ratio of the size of the BCI’s for Data IV 20-kernel weight and Data I is 0.16, and for Data IV days to anthesis and Data II is 0.33. Thus, the accuracy using meta-analysis of multiple-trait QTL and QTL identified through GWAS appears to be similar when the type of traits measured are similar. However, precision is better with the latter, which likely reflects the significantly larger number of QTL in the Data IV sets.

Despite the significantly greater resolution of the GWAS data sets, both distributions produced from Data IV displayed a gap around zero: within ±0.06 for 20-kernel weight and within ±0.16 for days to anthesis. Bennewitz and Meuwissen [1] discussed the potential drawbacks of using meta-analyses of QTL effects detected in bi-parental populations to characterize distributions of gene effects. A primary concern was for failure to identify all true QTL within the identified QTL sets either because certain alleles were excluded from the data set or because of a lack of statistical power to detect smaller-effect QTL [27, 28]. Even in cases where marker density and genome resolution is high, all QTL for a trait of interest may not be identified if mapping methods are utilized that screen detected effects against a significance threshold which basically excludes most of the small effect QTL. Furthermore, in genomic selection, the particular statistical method used to ‘train’ the model can influence the distribution of effects. Despite the higher resolution and multi-allelic nature of the Data IV data sets, the inability to detect QTL of near-zero effect was apparent, yet not problematic in fitting a distribution. Clearly, the distributions are centered at zero with highest frequencies observed in the zero vicinity and recognized as single-component. Thus, the results obtained with the GWAS data sets demonstrate the robustness of the DPGMM method with use of either meta-analysis of QTL identified across traits or large-scale data sets comprised of QTL for a single trait. If enough data are available, e.g. GWAS dataset, distributions of QTL effects based on a single trait rather than across traits might be more appropriate and useful. And with the advent of genotype-by-sequence (GBS) and other genotyping technologies that facilitate high-resolution marker sets, very dense marker sets are more available and more widely utilized in QTL identification [29]. For our purpose in exploring the impact of backcrossing strategies on recovery of performance of the hybrid targeted for conversion [8], the meta-analysis involving the Data I set based on a bi-parental population met our objectives well in that it focused on yield and yield component traits and additionally included other key traits essential to performance recovery i.e. maturity and abiotic stress tolerance.

### Distribution of QTL dominance coefficients

**Fig. 5:**
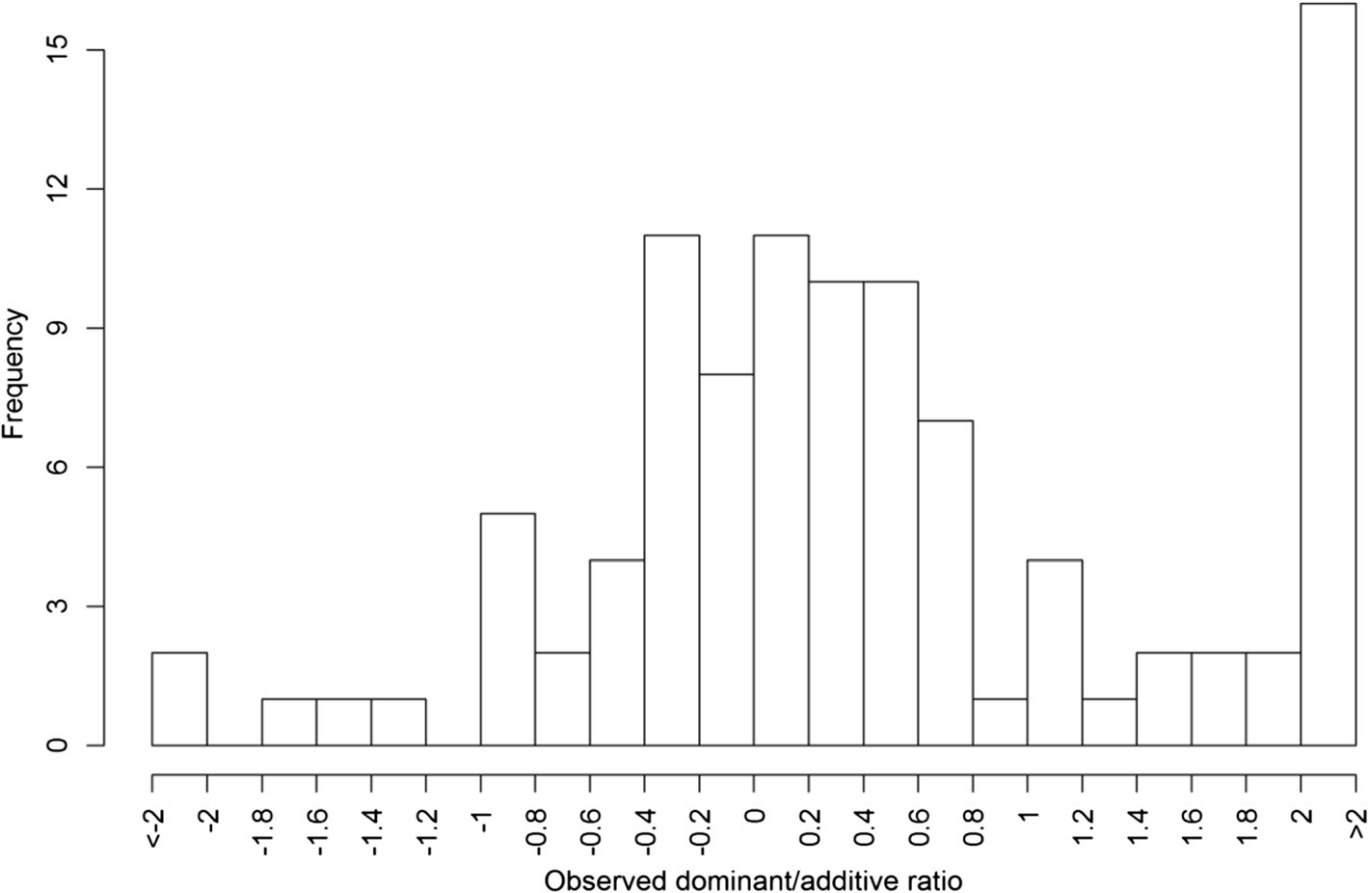
Histogram of observed dominance coefficients from meta-analysis based on five mapping populations

Observed dominance coefficients obtained from meta-analysis of five mapping studies varied in magnitude from less than -2.0 to more than 2.0 (Fig. 5), suggesting that all classes of dominance were represented among the traits measured. Around 50 % of the QTL (50 out of 101) displayed an *d/a* ratio in the range of -0.5 to 0.5, indicating partial recessivity, additivity, and partial dominance gene action. Approximately 25 % of the QTL exhibited either partially dominant or dominant gene action (0.5 < *d/a* < 1.25) or partially recessive or recessive gene action (-1.25 < *d/a* < -0.5). Furthermore, 25 % of the QTL exhibited apparent overdominance (>1.25) or underdominance (< -1.25) gene action.

**Fig. 6:**
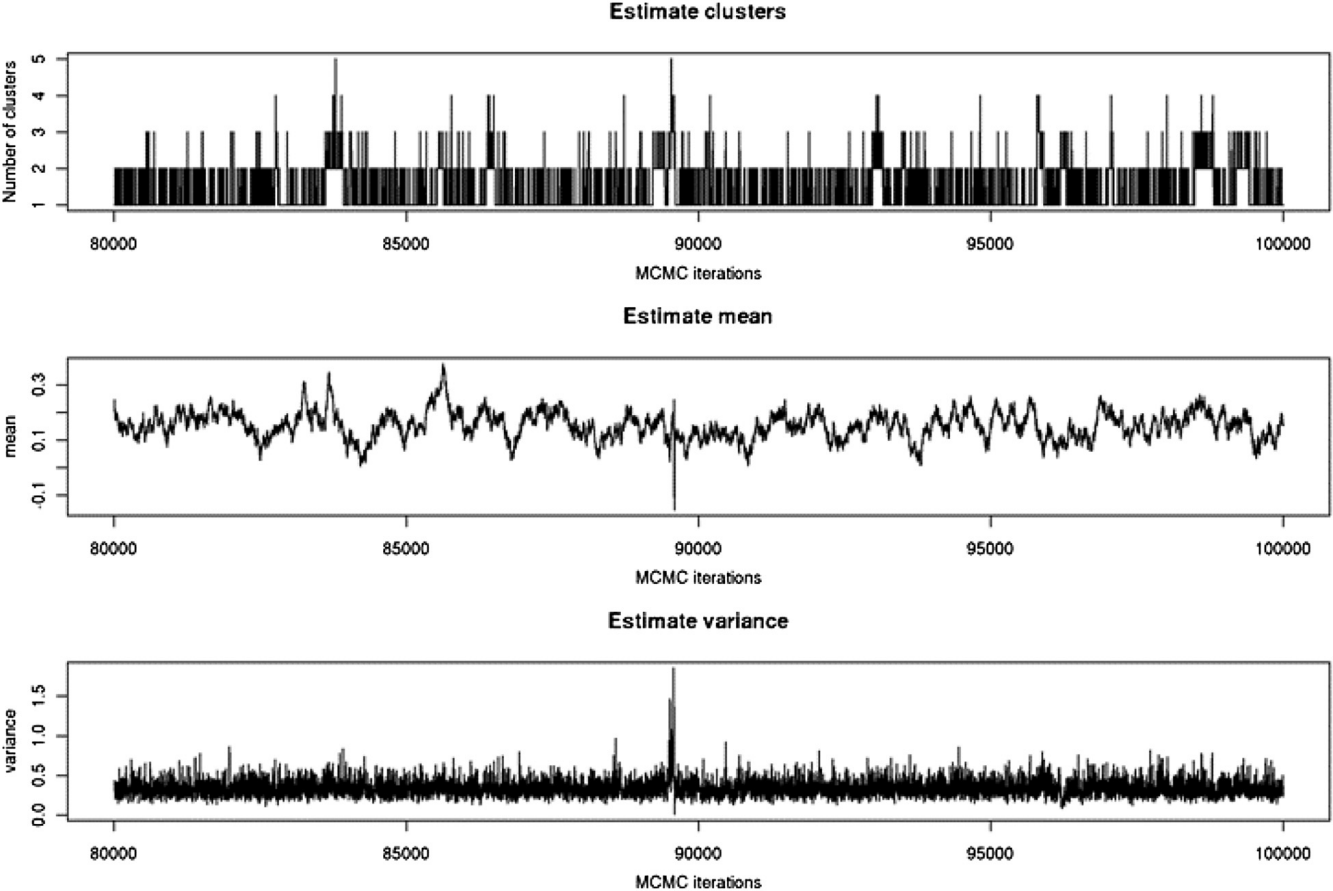
Estimation of cluster number, mean, and variance of the fitted distribution of dominance coefficients through MCMC

**Fig. 7:**
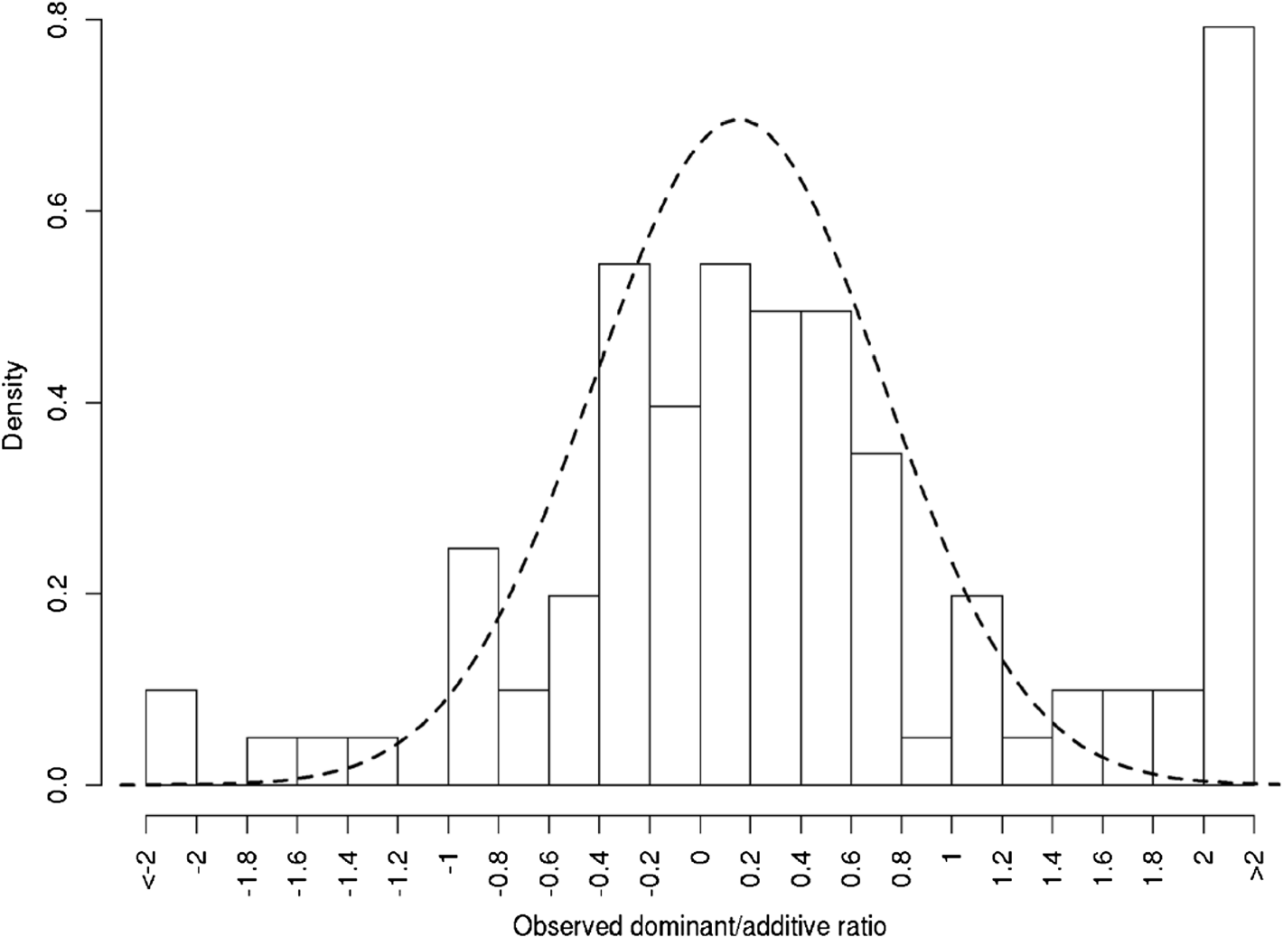
Normal distribution fitted to the dominance coefficients, with estimated mean at 0.152 with 95 % Bayesian confidence interval to be 0.055 and 0.237 and estimated variance at 0.329 with 95 % Bayesian confidence interval to be 0.193 and 0.542

Dominance coefficients were fitted with the normal distribution using DPGMM. The mode of posterior distribution with regard to K is 1, suggesting that all data could be fitted to a single component (Fig. 6); estimates of the distribution mean and variance were provided by MCMC as well. Figure 7 displays the estimated distribution overlaid on the density plot of observed data. The estimated mean of the distribution was 0.152 with 95 % BCI of 0.055 to 0.237, and variance of 0.329 with 95 % BCI of 0.193 to 0.542 (Table 4). The result that the distribution of dominance coefficients was fitted to a normal distribution with a positive mean conformed to previous studies [1].

## Conclusions

The DPGMM method offers an alternative to the over-simplified infinitesimal model in computer simulation as a means to better represent the genetic architecture of quantitative traits, which likely involve some large effects in addition to many small effects. Furthermore, it confers an advantage over other methods in that the number of mixture model components need not be known *a priori*. The DPGMM method takes advantage of prevalent QTL data to approximate the distributions of additive and dominance gene effects. The DPGMM method is robust with use of either meta-analysis of smaller-scale QTL analyses involving a number of traits or large-scale, single-trait QTL data sets. Furthermore, QTL data sets from bi-allelic or multi-allelic populations can be utilized. Although the data sets may be missing some near-zero QTL effects that were not resolved in the fundamental analyses, the methodology is able to accommodate this drawback. R code to facilitate use of the DPGMM method is included in a Additional file 1 to this paper (see section below).

The distributions of QTL additive and dominance effects highlighted through this study were used in modelling the genetic architecture of grain yield and other key performance traits for computer-simulated explorations to identify optimal breeding strategies to facilitate introgression of multiple value-added traits into an elite maize hybrid. Maize grain yield is a complex trait involving dominant and over-dominant gene action. Other traits important to recovery of the essential performance attributes of the hybrid targeted for conversion include maturity, resistance to lodging, and abiotic stress tolerance and these have a bearing on yield performance as well. Readers are directed to the recent work of Sun and Mumm [8] for an example of how the DPGMM-estimated genetic distribution parameters were deployed in computer simulations to evaluate breeding strategies.

## Appendix

Derivations of the fully conditional posterior distributions are given as follows:

$$ \begin{array}{l}P\left({\theta}_i\Big| else\right)\propto N\left({y}_i;{\theta}_i;{\sigma}_k^2\right)N\left({\theta}_i;{\mu}_k;{\tau}_i^2\right)\\ {}\propto \exp \left(-\frac{1}{2}*\frac{{\left({y}_i-{\theta}_i\right)}^2}{\sigma_k^2}\right)*\left(-\frac{1}{2}*\frac{{\left({\theta}_i-{\mu}_k\right)}^2}{\tau_i^2}\right)\\ {}\propto \exp \left(-\frac{1}{2}*\left(\frac{{\left({\theta}_i-{y}_i\right)}^2}{\sigma_k^2}+\frac{{\left({\theta}_i-{\mu}_k\right)}^2}{\tau_i^2}\right)\right)\\ {}\propto N\left({\theta}_i;\frac{\frac{y_i}{\sigma_k^2}+\frac{\mu_k}{\tau_i^2}}{\frac{1}{\sigma_k^2}+\frac{1}{\tau_i^2}},\frac{1}{\frac{1}{\sigma_k^2}+\frac{1}{\tau_i^2}}\right)\end{array} $$

$$ \begin{array}{l}P\left({c}_i=k\Big| else\right)\propto N\left({y}_i;{\theta}_i;{\sigma}_k^2\right)N\left({\theta}_i;{\mu}_k;{\tau}_i^2\right)p\left({c}_i\Big|{\mathbf{c}}_{-i},\alpha \right)\\ {}\propto \frac{1}{\sqrt{2\pi {\sigma}_k^2}} \exp \left(-\frac{{\left({y}_i-{\theta}_i\right)}^2}{2{\sigma}_k^2}\right)*\ \frac{1}{\sqrt{2\pi {\tau}_i^2}} \exp \left(-\frac{{\left({\theta}_i-{\mu}_k\right)}^2}{2{\tau}_i^2}\right)*{n}_{-i,k}\kern2em \\ {}=\frac{n_{-i,k}}{2\pi \sqrt{\sigma_k^2{\tau}_i^2}} \exp \left(-\frac{{\left({y}_i-{\theta}_i\right)}^2}{2{\sigma}_k^2}-\frac{{\left({\theta}_i-{\mu}_k\right)}^2}{2{\tau}_i^2}\right),\end{array} $$

$$ \begin{array}{l}P\left({c}_i=K+1\Big| else\right)\propto \alpha {\displaystyle \iint N\left({y}_i;{\theta}_i;{\sigma}_{K+1}^2\right)}N\left({\theta}_i;{\mu}_{K+1};{\tau}_i^2\right)N\left({\mu}_{K+1};{\mu}_0;{\sigma}_0^2\right)IG\left({\sigma}_{K+1}^2;{r}_1;{r}_2\right)d{\mu}_{K+1}d{\sigma}_{K+1}^2\\ {} \propto \alpha {\displaystyle \int N\left({y}_i;{\theta}_i;{\sigma}_{K+1}^2\right)IG\left({\sigma}_{K+1}^2;{r}_1;{r}_2\right)d{\sigma}_{K+1}^2}{\displaystyle \int N\left({\theta}_i;{\mu}_{K+1};{\tau}_i^2\right)}N\left({\mu}_{K+1};{\mu}_0;{\sigma}_0^2\right)d{\mu}_{K+1}\kern6.5em \end{array} $$

The first integration part:

$$ \begin{array}{l}{\displaystyle \int N\left({y}_i;{\theta}_i;{\sigma}_{K+1}^2\right)IG\left({\sigma}_{K+1}^2;{r}_1;{r}_2\right)d{\sigma}_{K+1}^2}\kern6.5em \\ {}={\displaystyle \int \frac{1}{\sqrt{2\pi }}{\left({\sigma}_{K+1}^2\right)}^{-{\scriptscriptstyle \frac{1}{2}}} \exp \left(-\frac{{\left({y}_i-{\theta}_i\right)}^2}{2{\sigma}_{K+1}^2}\right)*\frac{{r_2}^{r_1}}{\varGamma \left({r}_1\right)}{\left({\sigma}_{K+1}^2\right)}^{-{r}_1-1} \exp \left(-\frac{r_2}{\sigma_{K+1}^2}\right)}d{\sigma}_{K+1}^2\\ {}={\displaystyle \int \frac{1}{\sqrt{2\pi }}\frac{{r_2}^{r_1}}{\varGamma \left({r}_1\right)}{\left({\sigma}_{K+1}^2\right)}^{-\left({r}_1+{\scriptscriptstyle \frac{1}{2}}\right)-1} \exp \left(-\frac{{\scriptscriptstyle \frac{1}{2}}{\left({y}_i-{\theta}_i\right)}^2+{r}_2}{\sigma_{K+1}^2}\right)*}d{\sigma}_{K+1}^2\\ {}=\frac{1}{\sqrt{2\pi }}\frac{{r_2}^{r_1}}{\varGamma \left({r}_1\right)}\frac{\varGamma \left({r}_1+\frac{1}{2}\right)}{{\left(\frac{1}{2}{\left({y}_i-{\theta}_i\right)}^2+{r}_2\right)}^{r_1+{\scriptscriptstyle \frac{1}{2}}}}{\displaystyle \int IG\left({\sigma}_{K+1}^2;{r}_1+{\scriptscriptstyle \frac{1}{2}},{\scriptscriptstyle \frac{1}{2}}{\left({y}_i-{\theta}_i\right)}^2+{r}_2\right)*}d{\sigma}_{K+1}^2\\ {}=\frac{1}{\sqrt{2\pi }}\frac{{r_2}^{r_1}}{\varGamma \left({r}_1\right)}\frac{\varGamma \left({r}_1+\frac{1}{2}\right)}{{\left(\frac{1}{2}{\left({y}_i-{\theta}_i\right)}^2+{r}_2\right)}^{r_1+{\scriptscriptstyle \frac{1}{2}}}}\end{array} $$

The second integration part:

$$ \begin{array}{l}{\displaystyle \int N\left({\theta}_i;{\mu}_{K+1};{\tau}_i^2\right)}N\left({\mu}_{K+1};{\mu}_0;{\sigma}_0^2\right)d{\mu}_{K+1}\kern6.5em \\ {}={\displaystyle \int \frac{1}{\sqrt{2\pi {\tau}_i^2}} \exp \left(-\frac{1}{2}\frac{{\left({\theta}_i-{\mu}_{K+1}\right)}^2}{\tau_i^2}\right)}\frac{1}{\sqrt{2\pi {\sigma}_0^2}} \exp \left(-\frac{1}{2}\frac{{\left({\mu}_{K+1}-{\mu}_0\right)}^2}{\sigma_0^2}\right)d{\mu}_{K+1}\\ {}={\displaystyle \int \frac{1}{\sqrt{2\pi {\tau}_i^2}}\frac{1}{\sqrt{2\pi {\sigma}_0^2}} \exp \left(-\frac{1}{2}\frac{{\left({\theta}_i-{\mu}_{K+1}\right)}^2}{\tau_i^2}-\frac{1}{2}\frac{{\left({\mu}_{K+1}-{\mu}_0\right)}^2}{\sigma_0^2}\right)}d{\mu}_{K+1}\\ {}=\frac{1}{\sqrt{2\pi {\tau}_i^2}}\frac{1}{\sqrt{2\pi {\sigma}_0^2}}{\displaystyle \int \sqrt{2\pi \frac{1}{\left({\scriptscriptstyle \frac{1}{\tau_i^2}}+{\scriptscriptstyle \frac{1}{\sigma_0^2}}\right)}}\left({\mu}_{K+1};\frac{\frac{\theta_i}{\tau_i^2}+\frac{\mu_0}{\sigma_0^2}}{\frac{1}{\tau_i^2}+\frac{1}{\sigma_0^2}},\frac{1}{\frac{1}{\tau_i^2}+\frac{1}{\sigma_0^2}}\right) \exp \left(-\frac{1}{2}\frac{{\left({\theta}_i-{\mu}_0\right)}^2}{\tau_i^2+{\sigma}_0^2}\right)}d{\mu}_{K+1}\\ {}= \exp \left(-\frac{1}{2}\frac{{\left({\theta}_i-{\mu}_0\right)}^2}{\tau_i^2+{\sigma}_0^2}\right)\frac{1}{\sqrt{2\pi {\tau}_i^2}}\frac{1}{\sqrt{2\pi {\sigma}_0^2}}\sqrt{2\pi \frac{1}{\left({\scriptscriptstyle \frac{1}{\tau_i^2}}+{\scriptscriptstyle \frac{1}{\sigma_0^2}}\right)}}\\ {}=\sqrt{\frac{1}{2\pi \left({\tau}_i^2+{\sigma}_0^2\right)}} \exp \left(-\frac{{\left({\theta}_i-{\mu}_0\right)}^2}{2\left({\tau}_i^2+{\sigma}_0^2\right)}\right)\end{array} $$

Together we have:

$$ \begin{array}{l}P\left({c}_i=K+1\Big| else\right)\\ {}=\frac{\alpha }{2\pi}\frac{{r_2}^{r_1}}{\varGamma \left({r}_1\right)}\frac{\varGamma \left({r}_1+\frac{1}{2}\right)}{{\left(\frac{1}{2}{\left({y}_i-{\theta}_i\right)}^2+{r}_2\right)}^{r_1+{\scriptscriptstyle \frac{1}{2}}}}\sqrt{\frac{1}{\left({\tau}_i^2+{\sigma}_0^2\right)}} \exp \left(-\frac{{\left({\theta}_i-{\mu}_0\right)}^2}{2\left({\tau}_i^2+{\sigma}_0^2\right)}\right)\end{array} $$

$$ \begin{array}{l}P\left({\mu}_k\Big|{\theta}_i\in {k}^{th} cluster, else\right)\propto {\displaystyle \prod_{i=1}^{n_k}N\left({\theta}_i;{\mu}_k;{\tau}_i^2\right)}N\left({\mu}_k;{\mu}_0;{\sigma}_0^2\right)\\ {}\propto \exp \left(-\frac{1}{2}{\displaystyle \sum_{i=1}^{n_k}\frac{{\left({\theta}_i-{\mu}_k\right)}^2}{\tau_i^2}}-\frac{1}{2}\frac{{\left({\mu}_k-{\mu}_0\right)}^2}{\sigma_0^2}\right)\\ {}\sim N\left({\mu}_k;\frac{{\displaystyle \sum_{i=1}^{n_k}\frac{\theta_i}{\tau_i^2}}+\frac{\mu_0}{\sigma_0^2}}{{\displaystyle \sum_{i=1}^{n_k}\frac{1}{\tau_i^2}}+\frac{1}{\sigma_0^2}},\frac{1}{{\displaystyle \sum_{i=1}^{n_k}\frac{1}{\tau_i^2}}+\frac{1}{\sigma_0^2}}\right)\end{array} $$

$$ \begin{array}{l}P\left({\sigma}_k^2\Big|{y}_i\in {k}^{th} cluster, else\right)\\ {}={\displaystyle \prod_{i=1}^{n_k}N\left({y}_i;{\theta}_i;{\sigma}_k^2\right)}IG\left({\sigma}_k^2;{r}_1;{r}_2\right)\\ {}\propto {\left({\sigma}_k^2\right)}^{-{\scriptscriptstyle \frac{n_k}{2}}} \exp \left(-\frac{1}{2}\frac{{\displaystyle \sum_{i=1}^{n_k}{\left({y}_i-{\theta}_i\right)}^2}}{\sigma_k^2}\right){\left({\sigma}_k^2\right)}^{-{r}_1-1} \exp \left(-\frac{r_2}{\sigma_k^2}\right)\\ {}={\left({\sigma}_k^2\right)}^{-\left({r}_1+{\scriptscriptstyle \frac{n_k}{2}}\right)-1} \exp \left(-\frac{\frac{1}{2}{\displaystyle \sum_{i=1}^{n_k}{\left({y}_i-{\theta}_i\right)}^2}+{r}_2}{\sigma_k^2}\right)\\ {}\sim IG\left({\sigma}_k^2;{r}_1+\frac{n_k}{2},\frac{1}{2}{\displaystyle \sum_{i=1}^{n_k}{\left({y}_i-{\theta}_i\right)}^2+{r}_2}\right)\end{array} $$

## Additional file

Additional file 1:
**R code for DPGMM.** (DOCX 16 kb)

## Abbreviations

BCI: Bayesian confidence interval
CRP: Chinese Restaurant Process
DPGMM: Dirichlet Process Gaussian Mixture Model
EM: expectation-maximization
GMM: Gaussian Mixture Model
GWAS: genome-wide association studies
K: cluster number
MCMC: Markov Chain Monte Carlo
NAM: Nested Association Mapping
QTL: quantitative trait locus/loci
SE: standard error
SNP: single nucleotide polymorphism
