# Supplementary material for: Method to represent the distribution of QTL additive and dominance effects associated with quantitative traits in computer simulation
Source: BMC Bioinformatics. 2016 Feb 6;17:73. doi: 10.1186/s12859-016-0906-z (PMC4744427; doi:10.1186/s12859-016-0906-z)
Supplement: Additional file 1: — R code for DPGMM. (DOCX 16 kb) [file 12859_2016_906_MOESM1_ESM.docx]

**Sun and Mumm – Method paper (BMC Bioinformatics): R code for DPGMM**

set.seed(8888)

n = 150

clu_t <- numeric(n)

######### generate pseduo data with cluster = 2 ##########

x<-numeric(n)

y<-numeric(n)

tao<- runif(n,0,0.01)

for(i in 1:n){

u<-runif(1)

if(u<0.8){

x[i]<-rnorm(1,mean=0,sd=0.2)

y[i] <- rnorm(1,x[i],sd=sqrt(tao[i]))

clu_t[i] <- 1

}

else{

x[i]<-rnorm(1,mean=0,sd=0.6)

y[i] <- rnorm(1,x[i],sd=sqrt(tao[i]))

clu_t[i] <- 2

}

}

y1 <- y[which(y > .1|y < -.1)]

hist(y1,100)

y <- y1

tao <- tao

n <- length(y)

## prior setting

alpha = 0.02 ## larger value for higher probability of large number of cluster

r1 = 1

r2 =0.01

mu0 = 0

sigma0 = 0.01

## initiation of parameters

niter = 100000

theta <- rnorm(n,0,1)

nlh <- numeric(niter)

nclus <- vector("list",niter)

nnclus <- vector("list",niter)

clus=rep(1,n)

uni_mu=0;

uni_sigma=0.01;

## gibbs sampling

for (g in 1:niter){

negloglikhood = 0

if(g/10000==round(g/10000,dig=0)) print(g)

for(i in 1:n){

theta_var <- 1/(1/uni_sigma[clus[i]]+1/tao[i])

theta_mean <- (y[i]/uni_sigma[clus[i]]+uni_mu[clus[i]]/tao[i])*theta_var

theta[i] <- rnorm(1,theta_mean,sqrt(theta_var))

}

for(i1 in 1:n){

P=NULL;

k=length(unique(clus[-i1]))

if(k!=length(unique(clus))){ ## determine if clus[i1] has only one data, if yes, delete clus[i1]

ilab = clus[i1]

uni_mu = uni_mu[-ilab]

uni_sigma = uni_sigma[-ilab]

id = (1:n)[clus>ilab]

clus[id] = clus[id]-1 ## all the id should be decreased by 1.

}

for(j in 1:k){

nj <- length((1:(n-1))[clus[-i1]==j])

Ptemp <- 1/sqrt(2*pi*uni_sigma[j])*1/sqrt(2*pi*tao[i1])*exp(-1/2*(y[i1]-theta[i1])^2/uni_sigma[j])*exp(-1/2*(theta[i1]-uni_mu[j])^2/tao[i1])*nj

P <- c(P,Ptemp)

}

Pnew <- (alpha/2*pi)*(r2^r1/gamma(r1))*(gamma(r1+1/2)/(1/2*(y[i1]-theta[i1])^2+r2)^(r1+1/2))*sqrt(1/(tao[i1]+sigma0))*exp(-1/2*(theta[i1]-mu0)^2/(tao[i1]+sigma0))

psum <- (sum(P)+Pnew);

P <- P/psum;

Pnew <- Pnew/psum;

clus[i1]=sample(1:(k+1),1,replace=TRUE,c(P,Pnew))

if(clus[i1]==(k+1)){

mmean <- (mu0/sigma0+theta[i1]/tao[i1])/(1/sigma0+1/tao[i1])

mvar <- 1/(1/sigma0+1/tao[i1])

uni_mu <-c(uni_mu,rnorm(1,mmean,sqrt(mvar)))

uni_sigma <- c(uni_sigma,1/rgamma(1,r1+1/2,r2+1/2*(y[i1]-theta[i1])^2))# default is rgamma(n,shape,rate)

}

}

####### do resampling unique values after MCMC ####### Neal 2000, algorithm 2

for (i2 in 1:length(uni_mu)){

nk <- (1:n)[clus==i2]

sumthetaratio <- sum(theta[nk]/tao[nk])

suminvtao <- sum(1/tao[nk])

prss <- crossprod(y[nk]-theta[nk])

mmean <- (mu0/sigma0+sumthetaratio)/(1/sigma0+suminvtao)

mvar <- 1/(1/sigma0+suminvtao)

uni_mu[i2] <- rnorm(1,mmean,sqrt(mvar))

uni_sigma[i2] <- 1/rgamma(1,r1+length(nk)/2,r2+1/2*prss)

}

####### calculate negative log likelihood ##########

for(i3 in 1:n){

temp <- log(uni_sigma[clus[i3]])+(y[i3]-theta[i3])^2/uni_sigma[clus[i3]]

negloglikhood = temp + negloglikhood

}

nlh[g] <- negloglikhood

nnclus[[g]] <- clus

nclus[[g]] <- rbind(uni_mu,uni_sigma)

}

burnin = 80000;

num.clus <- NULL

for(i in 1:niter){

num.clus <- c(num.clus,length(unique(nnclus[[i]])))

}

barplot(table(num.clus[-(1:burnin)]),xlab="Cluster size")

plot(seq(from=burnin+1,to=niter,by=1),num.clus[-(1:burnin)],type="l", main="Estimate clusters",ylab="Number of clusters",xlab="MCMC iterations")

bmp("curve.bmp")

hist(y,n,freq=F,xlab="Additive effect")

curve(dnorm(x,mean=0,sd=sqrt(0.18)),add=T)

curve(dnorm(x,mean=0,sd=sqrt(0.09)),add=T)

dev.off()

###########################################

############ estimate posterior mode for cluster = 2

mu1 = NULL

mu2 = NULL

sigma1 =NULL

sigma2 =NULL

tempclus = NULL

index <- which(num.clus==2)

for(i in index[index>burnin]){

mu1 <- c(mu1,nclus[[i]][1,1])

sigma1 <- c(sigma1,nclus[[i]][2,1])

mu2 <- c(mu2,nclus[[i]][1,2])

sigma2 <- c(sigma2,nclus[[i]][2,2])

tempclus <- rbind(tempclus,nnclus[[i]])

}

mean(mu1) ### posterior mode

mean(sigma1) #### posterior mode

mean(mu2) ### posterior mode

mean(sigma2) #### posterior mode

hhh <- apply(tempclus,2,function(x)names(which.max(table(x)))) ## posterior mode for cluster

########### Bayesian confidence interval

hh <- apply(temp5,1,function(x){length(which(x==1))})

quantile(hh,c(0.025,0.975))
